# An evaluation of different classification algorithms for protein sequence-based reverse vaccinology prediction

**DOI:** 10.1371/journal.pone.0226256

**Published:** 2019-12-13

**Authors:** Ashley I. Heinson, Rob M. Ewing, John W. Holloway, Christopher H. Woelk, Mahesan Niranjan

**Affiliations:** 1 Faculty of Medicine University of Southampton, Southampton, United Kingdom; 2 Department of Biological Sciences University of Southampton, Southampton, United Kingdom; 3 Faculty of Medicine, University of Southampton, Southampton, United Kingdom; 4 Merck Exploratory Science Center, Cambridge, United States of America; 5 Department of Electronics and Computer Science, University of Southampton, Southampton, United Kingdom; UMR-S1134, INSERM, Université Paris Diderot, INTS, FRANCE

## Abstract

Previous work has shown that proteins that have the potential to be vaccine candidates can be predicted from features derived from their amino acid sequences. In this work, we make an empirical comparison across various machine learning classifiers on this sequence-based inference problem. Using systematic cross validation on a dataset of 200 known vaccine candidates and 200 negative examples, with a set of 525 features derived from the AA sequences and feature selection applied through a greedy backward elimination approach, we show that simple classification algorithms often perform as well as more complex support vector kernel machines. The work also includes a novel cross validation applied across bacterial species, i.e. the validation proteins all come from a specific species of bacterium not represented in the training set. We termed this type of validation Leave One Bacteria Out Validation (LOBOV).

## Introduction

**Fig 1:**
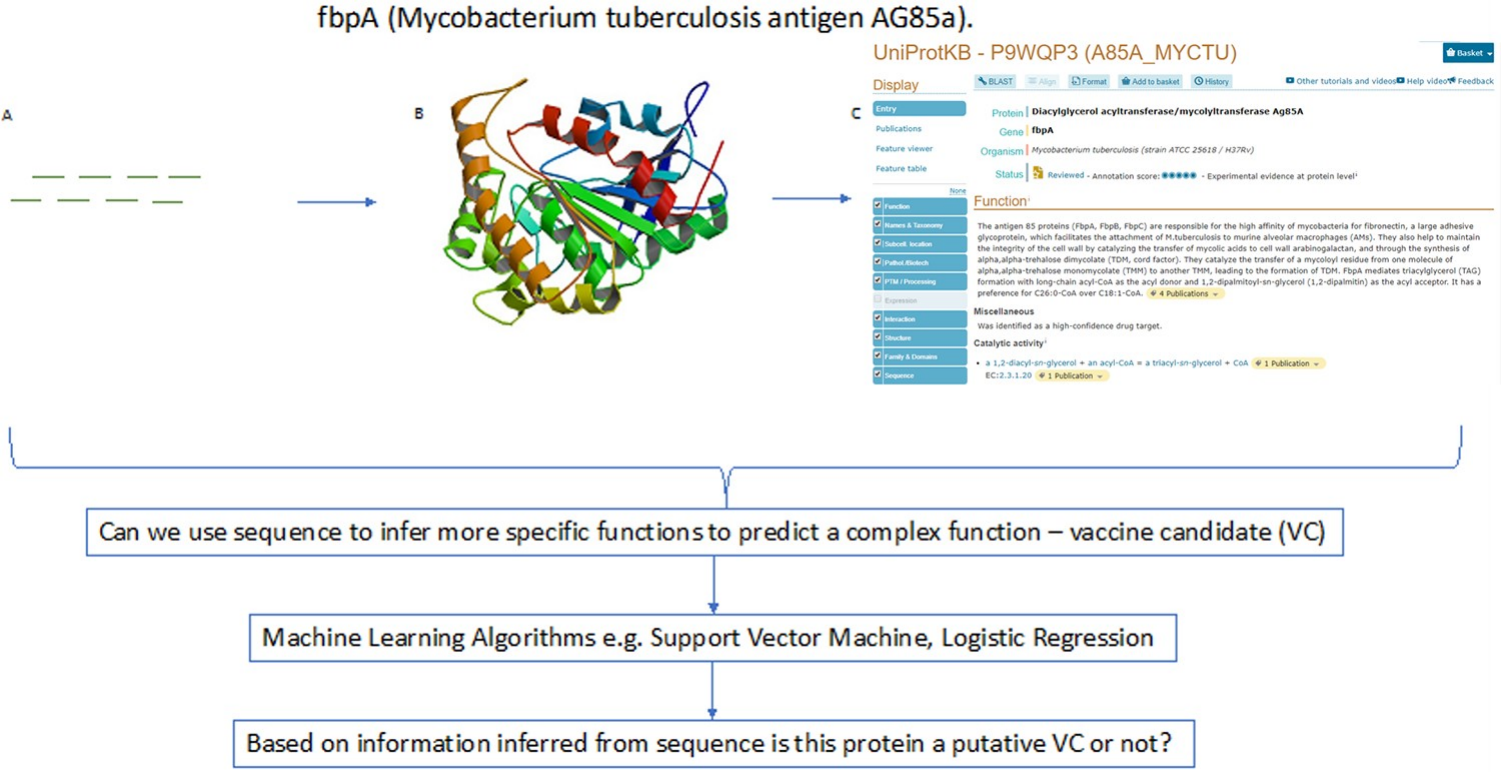
Depiction of the theory, can sequence infer function? The known vaccine candidate for mycobacterium tuberculosis[3] has many stages at which this can be evaluated, (a) the sequence, (b) structure[4] and (c) biological information[5] levels. The sequence information of proteins is the easiest information to obtain about proteins and therefore if we can predict high level biological information about a protein as a whole from a protein sequence data it can be extremely powerful. We apply the theory of sequence inferring function to vaccinology which utilized machine learning to make a prediction from sequence on the proteins function as a putative vaccine candidate.

The most biologically informative information about a protein is it’s function. In terms of sheer numbers many more proteins have a known sequence than we have evaluated their function (Fig 1). It has been suggested that we have only been able to detect 10% of all protein species in the laboratory[1], this severely limits our ability to accurately explore the proteome in our studies of human biology. Since we can very easily predict protein sequence (using the amino acid code) an ability to take the protein sequence and predict a protein’s function would dramatically improve the ability to study the human proteome. As an example, it has been estimated that there exists up to millions of protein types / isoforms within the human proteome[1], where as we have only determined the function of 20,316 human proteins[2]. It is with this in mind that we want to explore the limits of sequence-based inference, how able are we to predict a proteins function from sequence?

In computational biology, inference of structure and function from sequences has long been of interest[6] because much of the genetically derived information is contained in the sequence of nucleotides and amino acids of DNA and proteins respectively. Extracting the hidden information has been attempted with a plethora of statistical models including Hidden Markov Models (HMM), stochastic context free grammars and various pattern classification methods. The bulk of the literature on this subject covers the prediction of a particular molecular property from a small window of molecular sequence. Examples include the prediction of secondary structure[7], subcellular localization[8, 9] and protein-protein interactions[10, 11]. Implementations of several such efforts are publicly available as tools with web interfaces. They are widely used to predict a particular property of interest in isolation of others. Some authors have attempted to combine a small number of sequence derived features in an integrative framework. For example, Wieser et al[12] show that predicted local secondary structures could be used to enhance the inference of remote homologous relationships. In the reverse vaccinology (RV) prediction work we report here, we integrate predictions from a large number of sequence-based prediction tools to infer a complex higher level biological function. Such a combination, while having the advantage of uniting different modelling paradigms at the level of individual features, will naturally be limited in performance by the accuracies of the individual tools we build on. Hence, our study aims to combine these tools to form a more complete biological understanding from sequence of a complex trait that certain proteins exert. The stimulation of the human immune system. This work looks at the ability of sequence-based inference on a field of great public health importance; vaccinology.

Vaccinology is the only branch of modern medicine to completely eradicate an infectious disease[13] but it has long been said that the “vaccine golden era” has passed[14], this paper implements a technique aimed at revitalizing vaccine research by harnessing the power of the computing revolution, Reverse Vaccinology (RV). RV is a branch of vaccine research that uses computational approaches to discover novel vaccine candidates for bacterial pathogens. To date there have been two branches of RV, filtering approaches and machine learning (ML) approaches[15]. For a complete overview of all published RV methodologies please see Heinson et al “The Promise of Reverse Vaccinology”[15]. This publication focuses on the ML approaches to RV that uses the sequence of a protein to determine it’s protective ability. Ultimately, we build upon the most recently published RV ML methodology[16]. The previously published approach to ML in RV generated a Bacterial Protective Antigen (BPA) sequence dataset containing information and sequences of 200 BPAs and 200 non-BPAs. The BPAs and non-BPAs sequences were annotated with bioinformatics tools to describe the biology / function of these proteins. BPAs were previously defined as “a whole protein that led to significant protection (p < 0.05) in an animal model (i.e., bacterial load reduction or survival assay) following immunization and subsequent challenge with the bacterial pathogen”[17]. After curating the largest published dataset for use in the field of RV (BPAD200) Heinson et al[16] went on to show that they were able to improve technically (i.e. removing overfitting, removing an artificial bias, increasing the size of the training data and increasing the features on which the classifier was trained) on the previous ML RV study (Bowman et al[17]) and that they were able to classify BPAs from non-BPAs with a support vector machine (SVM) classifier that obtained an area under the curve (AUC) of 0.787. This previous work proved that a biological signal for protection (protective antigens) could be obtained from a literature curation[16]. In the previous work of Bowman et al[17] in this field we utlised linear regression as a classifier to show that SVM out performs more simple algorithms. However, linear regression is not a good choice for classifying a binary outcome. In this work we utilized logistic regression instead, which specifically works with two outcomes.

Proving that sequence data can successfully identify BPAs remains very timely given the rise of global antibiotic resistance. An example of the scale of this problem is that The World Health Organization currently list antibiotic resistance as one of the biggest threats to global health, food security and development[18]. Further to this, In the United States alone, two million people are infected with antibiotic resistant bacteria per annum. Antibiotic resistant infections are estimated to directly cost the US healthcare system in excess of $20 billion[19]. This growing resistance has been slow to be acted upon with funding bodies eager to fund therapeutics rather than preventative research[13]. However, there is now a wider acceptance that vaccinology offers one of the most cost effective and realistic chances for controlling infectious diseases and it was with this in mind that we explored the sequence dataset published previously for use in RV. This paper implemented different classification algorithms and showed that complex isn’t always best, thus improving the ease with which, and speed at which protein sequence data can be implemented for use in RV. Finally, this paper introduces a novel validation method for describing how RV ML classifiers can perform when predicting BPAs for species of bacterial pathogens not included within their training data (i.e. novel emerging bacterial pathogens). This novel validation was termed Leave One Bacteria Out Validation (LOBOV).

## Methods

### 2.1 Training dataset

This study utilised a previously assembled dataset of 200 Bacterial Protective Antigens (BPAs) and 200 non-BPAs as previously described [16]. Briefly, a BPA was defined as a bacterial protein that has led to significant protection (*p* <0.05) in an animal model following immunization and subsequent challenge with the bacterial pathogen. The Non-BPAs, negative training data, was generated by randomly selecting a protein from the same bacterial species and subcellular localization as each BPA. Additionally non-BPAs were discarded and re-sampled if (using BLASTP[20]) they matched to a BPA with > 98% similarity. Following this, to ensure the generation of a wide population of negative training data, non-BPAs were resampled if they matched to an already selected non-BPA with an E value > 10x10^-3^. It is important to create as diverse as possible non-BPA dataset. Even with the current E value filter it is limiting the generation of the non-BPA dataset in that one of the non-BPAs was not able to obtain the same subcellular localization as the matching BPA from the same bacterial species and was drawn from proteins with an unknown subcellular localization (still the same species though). This process is completely detailed in Heinson et al [16]. The final selection of 200 BPAs and 200 non-BPAs was then annotated to generate 525 features on which machine learning (ML) was used to classify BPAs and non-BPAs. These features were generated using 34 protein annotation tools which described biological properties of these proteins ranging from post-translational modifications such as methylation, glycosylation and acetylation to generic measurements such as amino acid percentage and hydrophobicity to epitope and MHC binding site predictions. Full details of how these features were generated and a complete list of the 525 features can be found in Heinson et al[16]. Heinson et al [16] termed their completed dataset BPAD200[16].

### 2.2 Comparison of machine learning classifiers

A comparison of different ML algorithms and their performance on the largest training dataset in reverse vaccinology (RV) (BPAD200) was undertaken. The previously defined ten best features (table 1 in Heinson et al[16]) were used to train each ML algorithm. The algorithms SVM, Logistic Regression and Random Forests were implemented in python, SciKit Learn[21]. Results were evaluated as AUCs generated using a leave tenth out cross validation (LTOCV) on the all BPAs and non-BPAs in the dataset BPAD200.

### 2.3 Greedy backward feature elimination and permutation analysis

Greedy backward feature elimination (GBFE) was implemented to obtain an optimum top ten features for logistic regression from the entire dataset (BPAD200, 525 features). This study was limited to utilising ten features as previously it had been shown that ten features achieved the greatest difference between biological signal from random noise in the dataset BPAD200[16]. To be able to asses how well the process of GBFE was able to select informative features permutation testing was undertaken. Ten features were selected at random from the complete dataset of 525 features and used to classify BPAs and non-BPAs. Classifier performances were evaluated using LTOCV to generate an average AUC. The process of selecting ten features and evaluating how they performed at classifying BPAs from non-BPAs was repeated 10,000 times to generate a frequency plot.

### 2.4 Leave one bacteria out validation (LOBOV)

The dataset of BPAD200 contained BPAs and non BPAs from 40 species of bacteria. To better evaluate the differences in predicting BPAs from specific species, even when not present in the training data we performed a new type of validation Leave One Bacteria Out Validation (LOBOV). Every bacterial species in the previously published dataset (BPAD200) that had > 4 examples of BPAs and non-BPAs combined in BPAD200 (n = 25) was removed sequenctially from the training dataset and used as a testing dataset. This process, LOBOV, enabled an understanding of how accurate ML in RV could be when predicting BPAs from unknown or bacterial pathogens that were not included in the training dataset.

## Results

### 3.1 Logistic regression performed equally as effectively when compared to more complex machine learning algorithms

**Fig 2:**
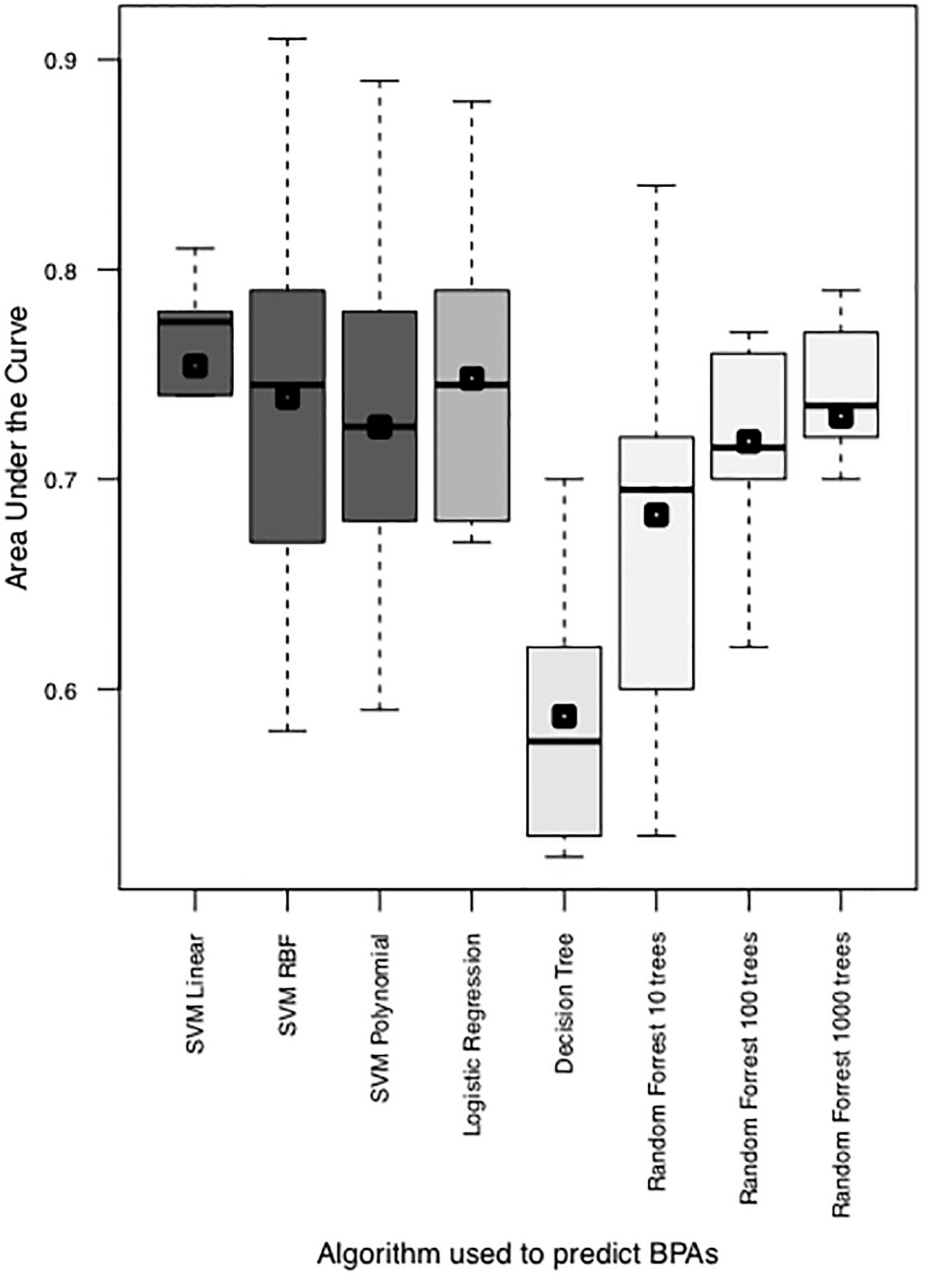
Comparison of area under the curve (AUCs) obtained in leave tenth out cross validation (LTOCV) of different machine learning algorithms predicting bacterial protective antigens (BPA) or non-BPAs from the curated dataset of BPAD200. All classifiers were built using the previously determined top 10 features for BPA prediction in reverse vaccinology for support vector machine (SVM) classification [16]. The mean of the LTOCV tenths were plotted as a square on the boxplot.

LTOCV was implemented to estimate the AUC on unseen data based on the training examples of BPAs and non-BPAs in BPAD200. Fig 2 visualizes the classification algorithm performances as boxplots representing the AUCs for each LTOCV when classifying BPAs and non-BPAs. It can be seen that the more “simple” algorithm of logistic regression obtains comparable accuracies to the more complex non-linear support vector machine (SVM) classification algorithms. These accuracies were obtained using features optimsed for SVM classification (Table 1 Heinson et al[16]). Informative features were determined using Greedy Backward Feature Elimination (GBFE) with a SVM and a radial bias function (SVM-RBF). It was hypothesized that utilizing the top ten most informative features established by GBFE with logistic regression would increase the AUCs obtained by logistic regression classifiers on BPAD200.

### 3.2 Pre-processing of the data impacted the ability to perform classification in reverse vaccinology

**Fig 3:**
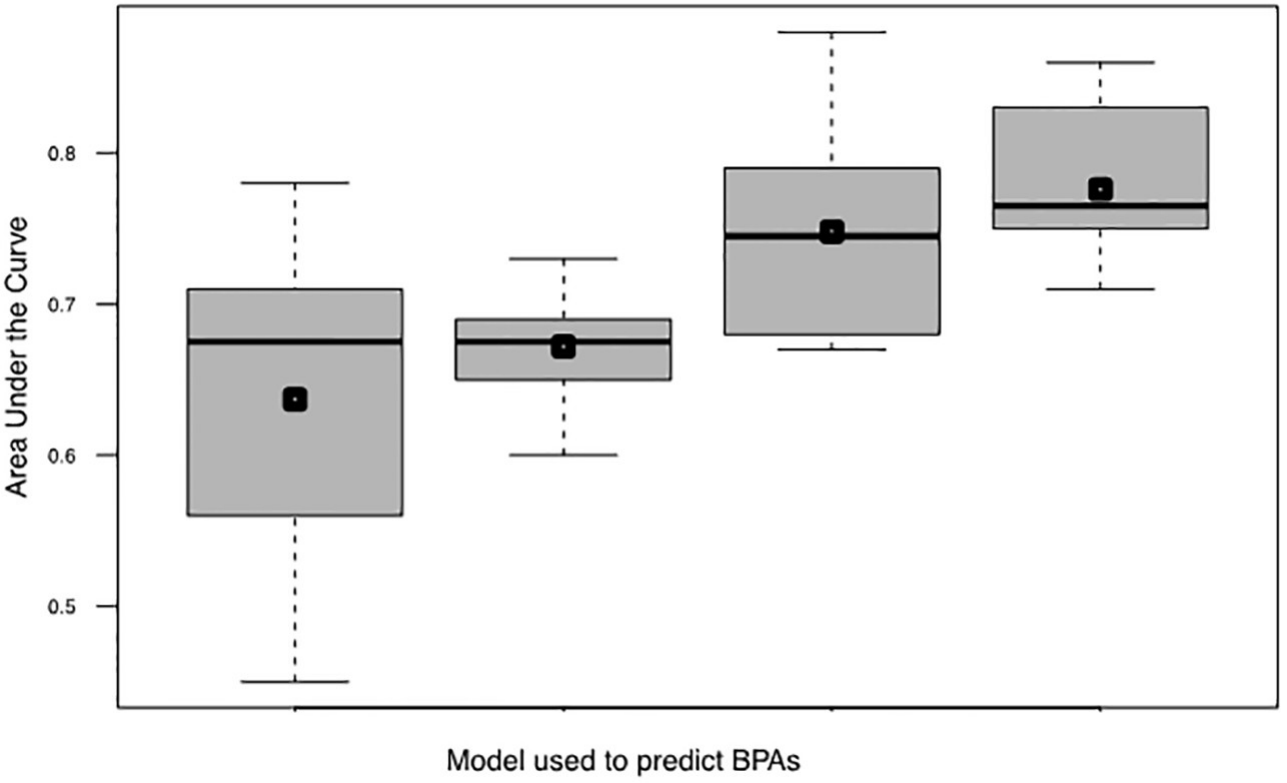
Comparison of how the pre-processing of the BPAD200 dataset impacts area under the curve (AUC) values obtained in reverse vaccinology (RV). Boxplots of AUC values were obtained by leave tenth out cross validation (LTOCV) of logistic regression classifiers predicting bacterial protective antigens (BPA) or non-BPAs from the curated dataset of BPAD200. From left to right; the raw dataset, the raw dataset scaled between -1 and 1 for each feature, using ten features scaled between -1 and 1 selected using GBFE with a support vector machine classifier as detailed in Heinson et al[16], using 10 features scaled between -1 and 1 selected using greedy backward feature elimination (GBFE) with a logistic regression classifier.

Fig 3 shows how pre-processing the data can impact classification accuracies obtained in RV. Using the raw data (all features not scaled) compared to data with features scaled between -1 and 1 improves the consistencies of AUCs for each LTOCV tenth. Next it was shown that implementing feature selection as described in Heinson et al[16] (GBFE using an SVM-RBF kernel) improves the AUCs obtained when classifying BPAs and non-BPAs (0.672 to 0.776 respectively). Finally, it was shown that implementing a GBFE that utilized logistic regression classifier improved the accuracies of logistic regression classifiers when compared to logistic regression classifiers trained using SVM GBFE derived features (0.739 to 0.776 mean AUC).

### 3.3 Greedy backward feature elimination and permutation analysis

**Fig 4:**
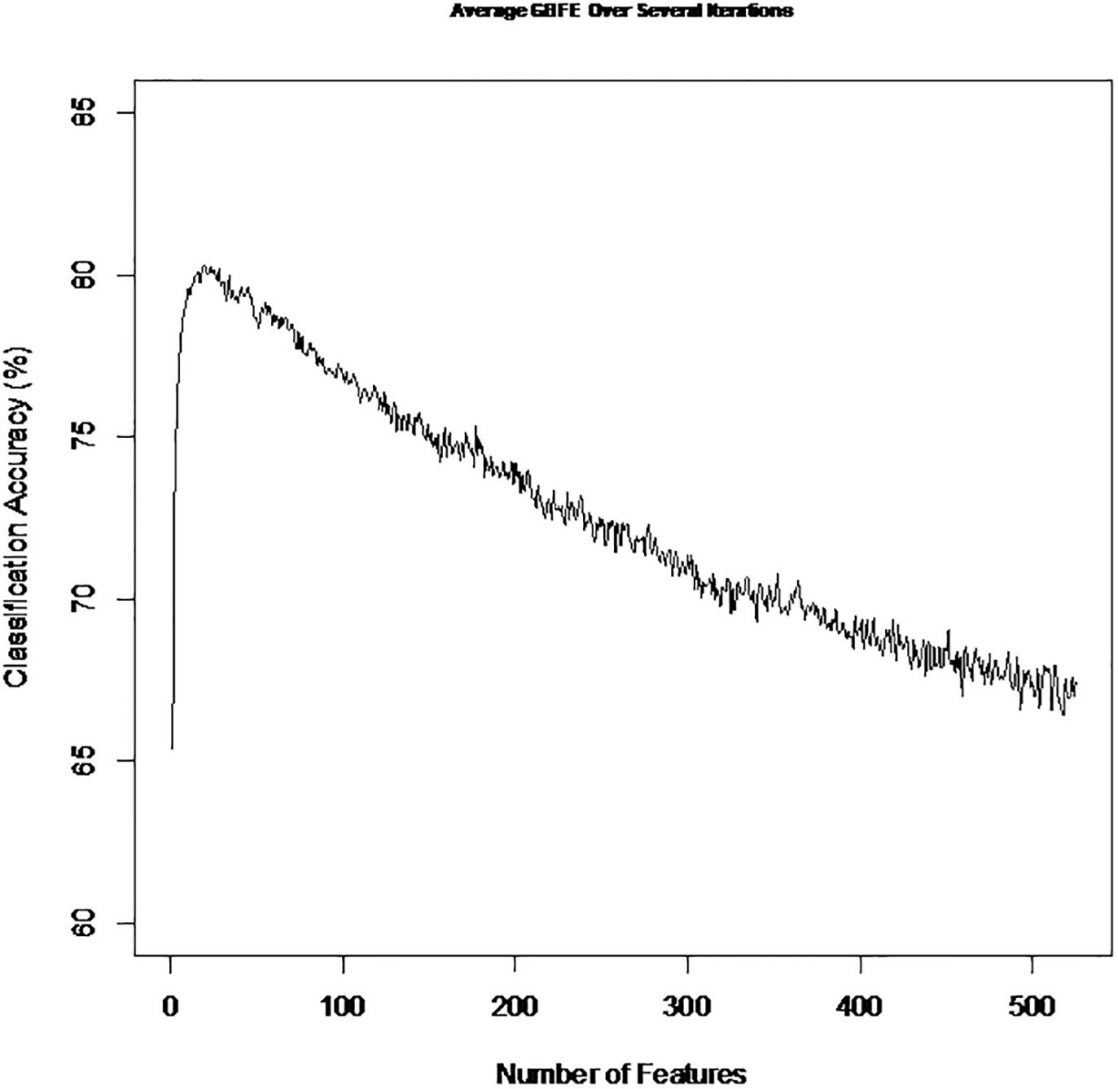
Accuracies obtained using a leave tenth out cross validation (LTOCV) to predict bacterial protective antigens (BPAs) or non-BPAs from the dataset BPAD200. Features were eliminated using a greedy backward feature elimination procedure. Black line depicts 10 feature cut off that was implemented in this study as it was shown for the dataset BPAD200 to best separate signal from noise[16].

Fig 4 shows that increasing the number of features used to predict BPAs from non-BPAs increases and as more noisy features are added the accuracy drops off. The main take away from the repeated iterations was the fact that in 9 out of 10 iterations the most informative feature was “MBAAgl7_CorCount” which is a feature derived from the protein annotation tool “GPS-MBA”[22] which predicts specific MHC class II epitope binding. This is very biologically relevant and gives confidence again that a biological signal of protection is being curated through the BPAD200 datasets generation method as Mhc II molecules are of vital importance in immunology and vaccine response[23, 24].

**Fig 5:**
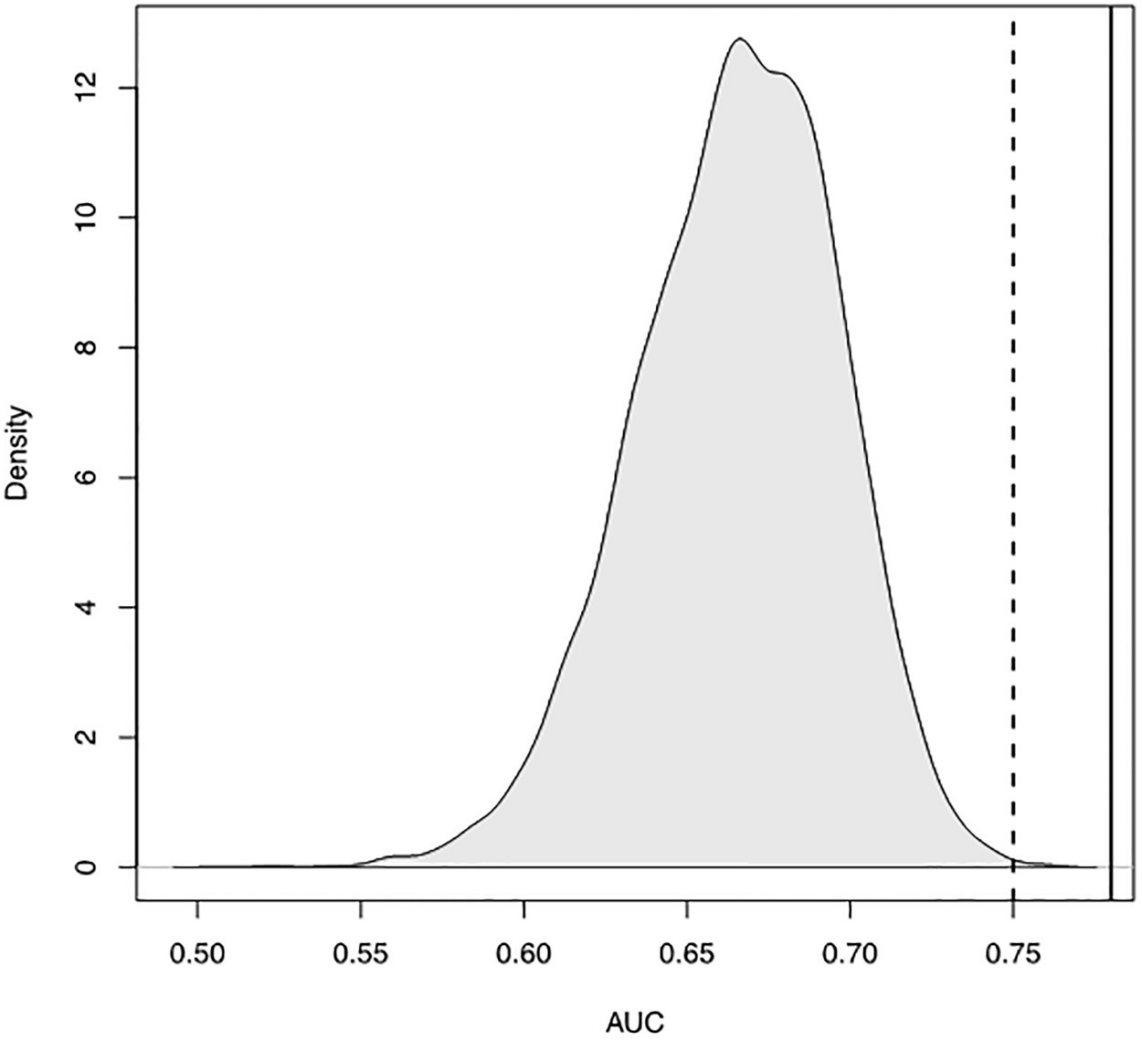
Area under the curve (AUC) obtained when classifying bacterial protective antigens (BPAs) from non-BPAs using a logistic regression classifier comprised of ten features from the dataset BPAD200. Ten features were selected randomly from the entire BPAD200 dataset (each feature was scaled between -1 and 1) and leave tenth out cross validation (LTOCV) with a logistic regression classifier was performed. This was repeated 10,000 times to obtain a frequency distribution. The dotted vertical line represents the AUC obtained through LTOCV when using the top ten features described previously in Heinson et al[16] with a logistic regression classifier. The solid vertical line is the AUC obtained from LTOCV using ten features derived from GBFE specifically of logistic regression.

By creating logistic regression classifiers comprised of ten random features from the BPAD200 dataset it was shown that GBFE strongly selects for the highest performing features (Fig 5 solid black line). By generating 10,000 logistic regression classifiers with ten random features each permutation from the BPAD200 dataset it was show that even when using a different classifier (SVM implemented by Heinson et al[16]) GBFE was still able to select strongly performing when used with a logistic regression classifier (Fig 5 dotted line).

### 3.4 Leave one bacteria out validation (LOBOV)

**Fig 6:**
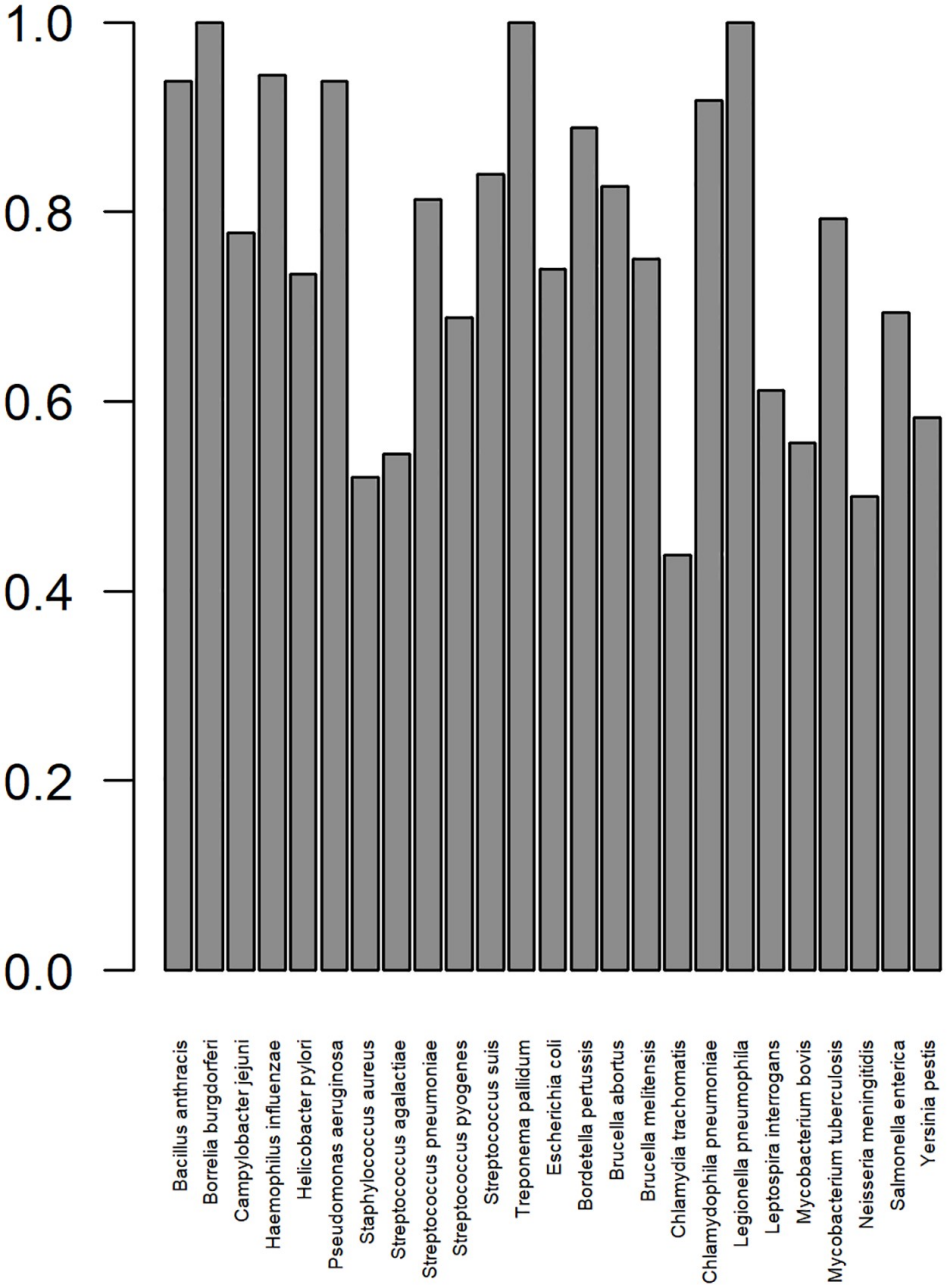
Area under the curve when predicting bacterial protective antigens (BPAs) and non-BPAs for each bacterial species that was left out of the training data and used as a test set, leave one bacteria out validation (LOBOV). All bacterial species in the dataset BPAD200 were left out of the training dataset to form a testing set in succession if there were > 4 examples of BPAs and non-BPAs combined in BPAD200 from that bacterial species.

A novel evaluation metric was implemented in this study titled LOBOV. LOBOV was implemented (Fig 6) and this showed that RV has the potential to make predictions of BPAs on bacterial species not present in the training dataset. However, the ability to classify BPAs and non-BPAs fluctuated dramatically across bacterial species. The result from this initial implementation of LOBOV has shown that RV may be much more beneficial when predicting BPAs for certain species of bacterial pathogens when compared to other species.

## Discussion

This study has built upon previous Machine Learning (ML) in Reverse Vaccinology (RV) methodologies and further explored the possibilities of sequence-based inference. Improvements to RV were achieved by further characterizing how multiple ML algorithms perform when implemented on the largest published sequenced based RV dataset (BPAD200). We have shown that ML in RV on the dataset of BPAD200 is not improved by using more complex algorithms such as support vector machines (SVM) and Random Forests and that the same area under the curve was achieved using logistic regression approaches (Fig 2). Despite what algorithm was selected the AUCs obtained when classifying BPAs and non-BPAs were comparable. The fact that more simple ML algorithms can be implemented with the same performance is a big key for implementing sequenced based RV practically. The purpose of RV is the prediction of proteins that can be included in novel vaccines and may represent a first line of defense to generate vaccines to newly emerging bacterial pathogens. To meet these demands when implementing RV on entire bacterial pathogens time is a critical factor and the ability to use more simple ML algorithms as effectively as more complex algorithms will speed up the process of RV being implemented and yielding useable novel BPAs.

When logistic regression classification of BPAs and non-BPAs was being explored it was also shown that how the data is pre-processed does impact the classifiers ability (Fig 3). Additionally, to data pre-processing we also showed the importance of isolating the most informative features (Fig 4). To identify informative features for logistic regression Greedy Backward Feature Elimination (GBFE) was implemented. As expected it was shown that using GBFE isolated the best set of features at a much better rate than by chance (Fig 5) It is proposed that for any RV ML approaches moving forward the preprocessing and feature selection is of vital importance when training and setting up the classifier to predict BPAs.

The most informative feature found by the GBFE for logistic regression classifiers of BPAs and non-BPAs was shown to be “MBAAgl7_CorCount”. This feature predicts Major histocompatibility complex (MHC) class II epitope binding sites. Mhc II responses have been shown very clearly to be of vital biological importance to vaccine stimulated immunity[24]. The main mechanism being through the degradation of proteins into peptides by professional antigen presenting cells which are then loaded onto the Mhc class II molecules. These peptides otherwise known as epitopes then stimulate CD4 T cells which control both the humoral and cellular branches of the immune system[25]. The most informative feature for the logistic regression classifiers supports previous publications assertions that a biological signal for protection is truly being captured from sequence information, generated by a literature curation when creating the dataset BPAD200[16]. As a point of interest, the top 10 features for sequence based logistic regression ML in RV are included in Table 1.

**Table 1:** The top ten most informative features as determined by greedy backward feature elimination on BPAD200 using logistic regression [26–35].

Tool	Refference	Tool Description	Accuracy Quoted in Original Publication
**DictyOGlyc**	26	The DictyOGlyc server produces neural network predictions for O-glycosylation	97% out of 39 test proteins
**NetGlycate**	27	NetGlycate 1.0 server predicts glycation of ε amino groups of lysines in mammalian proteins.	Matthews correlation coefficient 0.58
**NetPhosBac**	28	PhosBac 1.0 server predicts serine and threonine phosphorylation sites in bacterial proteins	0.74 AUC
**ProtParam**	29	ProtParam is a tool which allows the computation of various physical and chemical parameters for a given protein stored in Swiss-Prot or TrEMBL or for a user entered protein sequence.	NA
**YinOYang**	30	The YinOYang WWW server produces neural network predictions for O-ß-GlcNAc attachment sites in eukaryotic protein sequences	Not Listed
**LipoP**	31	The LipoP 1.0 server produces predictions of lipoproteins and discriminates between lipoprotein signal peptides, other signal peptides and n-terminal membrane helices in Gram-negative bacteria. Note: Although LipoP 1.0 has been trained on sequences from Gram-negative bacteria only, the following paper reports that it has a good performance on sequences from Gram-positive bacteria also	96.8% Gram Negative 92.9% Gram Positive
**NetOGlyc**	32	The NetOglyc server produces neural network predictions of mucin type GalNAc O-glycosylation sites in mammalian proteins.	83% glycosylated and 90% of the non-glycosylated
**PSORTb**	8	Bacterial Subcellular Localization predictor	Matthew correlation coefficient 0.79 Gram Positive, 0.85 Gram Negative
**GPS-CCD**	33	Calpains Ca2+ dependent cysteine proteases control numerous biological processes, gene expression, cell death, cell cycle progression. Predicts calpain cleavage motifs in proteins.	Matthew Correlation Coeficient 0.0908, Accuracy 89.98%. Medium setting as implimented.
**NetMhcPan**	34	NetMHCpan server predicts binding of peptides to any known MHC molecule using artificial neural networks (ANNs).	Only look at the sensitivity. AUC as 0.92 in the PickPocket Paper [35].

It was hypothesized that the overall accuracy of the bioinformatic tools implemented to derive biological information from this sequence would be a limiting factor to ML RV approaches. However, as can be seen some of the bioinformatic tools do obtain very high accuracies when classifying a specific biological characteristic. The fact that the AUC of the individual tools is higher than the AUC obtained by utilizing these tools to predict BPAs (0.78 average AUC of LTOCV) can be linked to the complexity of using protein annotation tools to predict protective antigens for RV approaches. ML in RV is making predictions on complex biological interactions, as opposed to specific sites, represented by pre-determined AA sequences. Moreover, the drop in accuracy when predicting BPAs compared to the individual tools should mostly be attributed to the fact that there is still no strict definition of what makes a protein a protective antigen and that a wide range of biological phenomena is thought to play a part in determining a protein’s immune stimulating ability[36].

Finally, this study implemented a novel evaluation metric, Leave One Bacteria Out Validation (LOBOV). LOBOV aimed to predict how well RV would perform when predicting potential BPAs for inclusion into vaccines for bacterial pathogens from which there are no known examples of BPAs. Examples of when there may be no BPAs documented are if there is a newly emerging undefined bacterial pathogen spreading through a population or a bacterial species that is unculturable in the laboratory [15]. The results from the LOBOV metric for the dataset BPAD200 (Fig 6) show that RV’s ability to predict effective BPAs for different bacterial species differ dramatically. Meaning that RV may be more beneficial when applied to certain organisms. It was envisaged that a biological reason for this would have been unearthed. Attributing a biological trait to the LOBOV results would have enabled RV to be focused on groups of bacteria that are more likely to have a positive outcome with the current approaches and further research to be directed at the currently harder to predict BPAs of certain bacterial species. Despite the underlying biology of the LOBOV results not being unearthed, this approach may still be able to target current RV approaches using ML. For example, one may be more confident in BPA predictions made for Bordetella pertussis as opposed to Chlamydia trachomatis.

The work detailed in this manuscript is an enhancement to the literature of sequence based ML in RV methodology and represents an important step in understanding how ML can be fully explored in the field of RV. It was shown that more simple algorithms perform just as well as more complex which means that the speed that RV can be applied at to make predictions of BPAs has been increased. Ultimately, what this work suggests is that placing the emphasis on building “better” and more complex algorithms, as is commonly done, is not overly helpful and future work should focus on the biological information that they are built upon.

## Conclusion

Due to the complexity of predicting vaccine candidates, Reverse Vaccinology (RV) is a great field in which to test sequence based inference. The amino acid sequence of a protein may contain sufficient information to make such predictions of potential vaccine candidates based on features that we derive from sequence. This work shows the limits of such prediction by utilising a dataset of 400 proteins, 525 sequence-based features and systematically applying a range of classification algorithms. We note that, given these rich features, simple classifiers (logistic regression) perform as well as, or marginally better than, more complex approaches (support vector machines with non-linear kernels) often used in the field of bioinformatics. We also show the importance of data pre-processing through scaling and feature selection. It was determined that greedy backward feature elimination performed very well when selecting informative features. Finally, we proposed and implemented a new metric to gauge how well RV classifiers perform on unknown “newly emerging” pathogens and this was termed leave One Bacteria Out Validation (LOBOV).
